# The Contribution of the Airway Epithelial Cell to Host Defense

**DOI:** 10.1155/2015/463016

**Published:** 2015-06-21

**Authors:** Frauke Stanke

**Affiliations:** ^1^Department of Pediatrics, Hannover Medical School, Carl-Neuberg-Strasse, 30625 Hannover, Germany; ^2^Biomedical Research in Endstage and Obstructive Lung Disease Hannover (BREATH), German Center for Lung Research, Hannover, Germany

## Abstract

In the context of cystic fibrosis, the epithelial cell has been characterized in terms of its ion transport capabilities. The ability of an epithelial cell to initiate CFTR-mediated chloride and bicarbonate transport has been recognized early as a means to regulate the thickness of the epithelial lining fluid and recently as a means to regulate the pH, thereby determining critically whether or not host defense proteins such as mucins are able to fold appropriately. This review describes how the epithelial cell senses the presence of pathogens and inflammatory conditions, which, in turn, facilitates the activation of CFTR and thus directly promotes pathogens clearance and innate immune defense on the surface of the epithelial cell. This paper summarizes functional data that describes the effect of cytokines, chemokines, infectious agents, and inflammatory conditions on the ion transport properties of the epithelial cell and relates these key properties to the molecular pathology of cystic fibrosis. Recent findings on the role of cystic fibrosis modifier genes that underscore the role of the epithelial ion transport in host defense and inflammation are discussed.

## 1. Introduction into the Defense Repertoire of the Epithelial Cell

The chloride and bicarbonate transporter CFTR, in healthy individuals as well as in F508del homozygous CF patients, is expressed on the apical surface of epithelial cells [1]. CFTR colocalizes with other ion channels such as the amiloride-sensitive epithelial sodium channel [2, 3]. The amount of liquid that covers the apical side of the epithelium is tightly regulated as the net ion transport through the apical membrane, driven by epithelial sodium and chloride channels, paralleled by water transport [3]. In turn, the amount and composition of the epithelial lining fluid determine the efficacy of mucociliary clearance [4], a mechanism by which the epithelium can detoxify pathogens and pollutants [4]. Host defense is mediated by resident macrophages that are localized on the epithelial surface [4] as well as by antimicrobial proteins that are secreted into the epithelial lining fluid [4]. The efficacy of mucociliary clearance depends on the beating of the epithelial cell's cilia [4] and on the viscoelastic properties of the fluid that covers the epithelium [4, 5]. Thereby, CFTR directly influences the extent to which the fluid on the airway's surface can be moved: firstly, chloride, and, in consequence, water, secreted via CFTR at the apical side of epithelial cells. Secondly, CFTR secretes bicarbonate, whereby the regulation of the pH determines whether secreted components such as mucins can unfold properly [6].

Apart from ion channels, the apical membrane of epithelial cells is equipped with a variety of receptors that sense the presence of pathogens or the inflammatory state: toll-like receptors that directly interact with bacterial or viral components [7, 8] as well as receptors for macrophage-derived cytokines such as TNF*α* [9] and IFN*γ* [10] are expressed by epithelial cells [7, 8, 11, 12]. Hence, the epithelial cell is well equipped to detect the presence of pathogens as well as the activity of macrophages that reside in the epithelial surface fluid. In other words, the airway epithelial cell is in a unique position to recognize the need for host defense as well as providing it via an activation of the chloride and bicarbonate channel CFTR.

## 2. Cytokines Alter the Ion Conductance Capabilities of Airway Epithelial Cells

In order to recognize that cytokines can alter the ion secretion properties of epithelial cells, two fields that are traditionally not well linked, that is, experimental immunology and electrophysiology, need to interact. Fortunately, several experiments wherein airway epithelial cells have been exposed to cytokines and the expression or function of ion channels such as CFTR, the amiloride-sensitive epithelial sodium channel ENaC, and calcium-activated chloride channels is monitored by comparative RT-PCR, western blot, or electrophysiology have been provided since the turn of the century ([15–25]; Table 1). Roughly summarized, the uptake of sodium by airway epithelial cells through ENaC is inhibited by TGF*β* [15, 25], IL13 [16, 22], IL1*β* [18], TNF*α* [19], IL4 [16], and IFN*γ* [17]. In contrast, the secretion of chloride and/or bicarbonate via CFTR is increased by IL17 [21], IL13 [16, 20], IL4 [16], and TNF*α* [17] and via the chloride transporter SLC26A9 by IL13 [23]. In conclusion, the data generated by independent researchers paints a highly coherent picture of the cross-talk between immunologically relevant cells and airway epithelial cells; cytokines, being released by immunologically active cells, will be interpreted by the epithelial cell as a signal to increase the epithelial surface fluid, thereby promoting mucociliary clearance and decreasing the amount of pathogens and inflammatory substances within the lung.

The clinical importance of these findings is underlined (a) by the susceptibility of CFTR-deficient individuals to nosocomial pathogens, as observed in cystic fibrosis, (b) by the susceptibility of ENaC-deficient patients who suffer from pseudohypoaldosteronism type I [26] to* P. aeruginosa* [27, 28], and (c) by the elevated susceptibility of patients with CF-like disease carrying partially dysfunctional CFTR and/or ENaC gene variants to respiratory disease [29]. Furthermore, the impaired regulation of lung fluid balance by the cytokine TGF*β* has now been recognized as a direct cause for acute respiratory distress syndrome [30].

## 3. Cystic Fibrosis Modifying Genes That Determine Immunology and Inflammation Alter the CFTR-Mediated Basic Defect 

**Figure 1:**
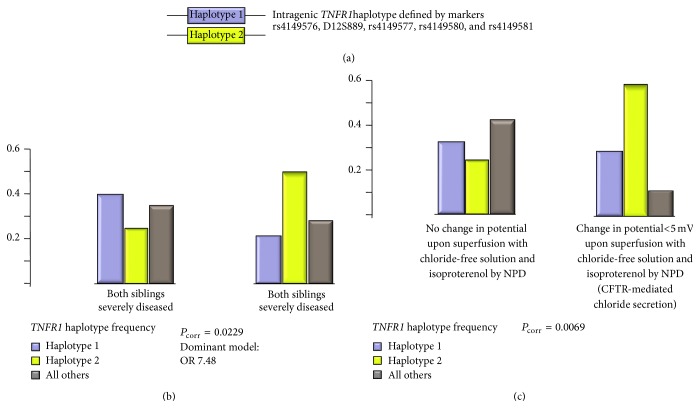
Association of* TNFR1* variants with CF disease severity and manifestation of CFTR-mediated residual chloride secretion in respiratory tissue among F508del-*CFTR* homozygous CF patients. (a) Definition of* TNFR1* variants. Two contrasting haplotypes, designated haplotype 1 and haplotype 2 within this figure, were described by typing the five markers rs4149576, D12S889, rs4149577, rs4149580, and rs4149581 among 101 families with a total of 171 F508del-*CFTR* homozygous CF patients [13]. Haplotypes were reconstructed using the software FAMHAP [14]. Association was judged by case-reference association whereby cases and references were defined based on the disease severity of the siblings (b) or the manifestation of a change in potential upon superfusion of the nasal epithelium with chloride-free solution and isoproterenol (c). All *P* values reported within this figure were calculated by FAMHAP and are corrected for sibling dependence and testing of multiple markers [14]. Please note that the case and control subpopulations compared within (b) and (c) were defined independently and were nonoverlapping (for details, please see [13]). Please also note that haplotype 2, depicted in green within this figure, is overrepresented among sib pairs with mild CF disease and among patients who display CFTR-mediated residual chloride secretion by NPD. As a causal interpretation, this might reflect the crosstalk between the cytokine pathway and the ion secretory properties of the epithelium [15–25], indicating that the mild* TNFR1* haplotype 2 is more susceptible to translating the action of the host defense modifier gene* TNFR1* into CFTR-mediated residual function. Alternatively, as CFTR-mediated residual chloride secretion causes a mild disease phenotype, the observed* TNFR1* association with the manifestation of the basic defect in NPD might reflect an overrepresentation of mild modifier alleles among patients with residual CFTR-mediated chloride secretion, which is equivalent to a replication study with confirmatory outcome.

Modifier genes of cystic fibrosis disease severity have now been studied for a decade [31, 32]. Many studies are candidate genes based; that is, the investigators rely on a hypothesis of which of the 22.000 protein-coding human genes [33] is likely to influence CF disease. Several researchers have selected genes encoding for cytokines such as* TNFA*,* IL1B,* and* TGFB1* as candidate genes because of their known role in infection, immunology, and inflammation [13, 34–38]. Among these immunologically relevant candidate genes,* IL1B* has been replicated in two truly independent studies [35, 39], albeit the molecular variant has not been mapped by the base yet. The cytokine receptors* TNFR1* and* IFNGR1* have been studied as modifier genes in* European CF Twin and Sibling Study* [13, 39, 40]. Until now, the CF basic defect that can be assessed by nasal potential difference measurement in vivo has only been used by the* European CF Twin and Sibling Study* for an association study. Strikingly, the TNF*α* receptor 1 gene* TNFR1* was observed as a modifier of CF disease severity as well as of CFTR-mediated residual chloride secretion in the nasal epithelium, whereby the risk and the benign allele were identified consistently for both traits ([13, 40], Figure 1). Furthermore, several immunologically relevant genes were identified as modifiers of the CFTR-mediated basic defect ([13], Table 2). This observation parallels the aforementioned observed capabilities of cytokines to activate fluid secretion by airway epithelial cells in order to promote clearance: as functional experiments using human airway epithelial cell lines, primary airway epithelial cells, and animal models have demonstrated that cytokines can alter ion and fluid transport in the respiratory epithelium effectively [15–25], it must be expected that genetic variants in cytokines and their receptors show genetic association with the manifestation of the basic defect among cystic fibrosis patients [13].

## 4. Outlook

Functional data and genetic evidence indicate that the epithelial cell and the immune system interact to regulate ion and fluid secretion. It remains to be clarified by which molecular mechanism this is accomplished within the epithelial cell. Likely, part of the effect of cytokines on ion transport will be mediated by the signal transduction cascade that is set into motion by the contact between the soluble ligand and the membrane-bound receptor. While a target of such regulatory networks might be the CFTR gene itself, it is equally plausible that the configuration of the epithelial cell is altered to promote more efficient trafficking of the CFTR ion channel, known for its short half-life [41] and its prolonged residence in a subapical compartment [42–44], to the apical membrane. So far, an association with the CF basic defect has been described for genes encoding the two transcription factors* STAT3* [39] and the epithelial-specific transcription factor* EHF* [45], the latter being a positional candidate that has been selected for replication based on a genome-wide study undertaken to identify modifiers of CF lung disease severity [46]. However, in order to effectively select therapeutic targets in the future, the central molecular pathways that are used by host defense modifier genes which have an impact on CFTR-mediated residual function or ENaC activity in epithelial cells need to be identified. Ultimately, a drug that interferes with the activity of key regulatory elements—such as regulatory microRNAs and transcription factors—which translate the action of host defense modifier genes into CFTR-mediated residual function or ENaC activity will be an attractive instrument to counterbalance the susceptibility of CF patients to infection and inflammation.

## Figures and Tables

**Table 1:** Effect of cytokines on ion transport in airway epithelial cells.

Model system	Cytokine	Ion channel	Techniques	Principle findings	Reference	EPub
Rabbit	TGF*β*	ENaC	^22^Na^+^ efflux, electrophysiology, flow cytometry, surface biotinylation, and RT-PCR	TGF*β* decreases ENaC-mediated sodium and fluid uptake	[25]	2014

Air liquid epithelial cell culture with human CFBE41o^−^ and transfected cells	IL6, IL8, and CXCL1/2	CFTR, TMEM16A	Electrophysiology, immunocytochemistry, human cytokine antibody array, and cell-surface ELISA	CFTR and TMEM16A decrease secretion of IL6, IL8, and CXCL1/2	[24]	2012

Slc26a9-deficient mice	IL13	Slc26a9	Electrophysiology	IL13 increases Slc26a9-mediated Cl^−^ secretion	[23]	2012

BALB/c mice	IL13	ENaC	Electrophysiology, RT-PCR	IL13 decreases ENaC expression	[22]	2010

Human bronchial epithelial cells	IL17A	Bicarbonate transport	Electrophysiology, measurement of surface and intracellular pH	IL17A increases HCO_3_^−^ secretion	[21]	2009

Human nasal epithelial cells	IL13	CFTR	Immunocytochemistry, western blot	IL13 increases CFTR expression	[20]	2007

Primary culture of rat alveolar epithelial type II cells, primary culture of human alveolar epithelial type II cells	Il1*β*	ENaC	^22^Na^+^ efflux, electrophysiology, western blot, and RT-PCR	IL1*β* decreases ENaC expression	[18]	2005

Rat alveolar epithelial cells	TNF*α*	ENaC	Electrophysiology, northern blot, and western blot	TNF*α* decreases ENaC expression	[19]	2004

Primary culture of rat alveolar epithelial type II cells, primary culture of human alveolar epithelial type II cells	TGF*β*	ENaC	^22^Na^+^ efflux, electrophysiology, western blot, and RT-PCR	TGF*β* decreases ENaC expression	[15]	2003

Primary culture of human bronchial epithelial cells	IL4, IL13	CFTR, ENaC	Electrophysiology, western blot	IL4 and IL13 increase CFTR expression IL4 and IL13 decrease ENaC expression	[16]	2002

Primary culture of human bronchial epithelial cells	IFN*γ*, TNF*α*	ENaC, CFTR	Electrophysiology, western blot, and transepithelial fluid transport	IFN*γ* decreases CFTR expression IFN*γ* decreases ENaC-mediated sodium transport TNF*α* increases CFTR expression	[17]	2000

**Table 2:** Immunologically relevant genes and transcription factors that were identified as modifiers of the CFTR-mediated basic defect among F508del-*CFTR* homozygous CF patients.

Functional category	Gene	Association observed with	Reference
Ligands	*CD14 *	CFTR activity/intestinal tissue [C] Non-CFTR mediated residual chloride secretion/intestinal tissue [D]	[13]
	*TNFA *	CFTR activity/nasal epithelium [B]	[13]
	*IL1B *	CFTR activity/intestinal tissue [C]	[13, 39]
	*TGFB1 *	Non-CFTR mediated residual chloride secretion/intestinal tissue [D]	[13]
	*IL8 *	CFTR activity/nasal epithelium [B]	[13]

Membrane-bound receptors	*TLR4 *	ENaC activity/nasal epithelium [A] CFTR activity/nasal epithelium [B] CFTR activity/intestinal tissue [C]	[13]
	*TNFR1 *	ENaC activity/nasal epithelium [A] CFTR activity/nasal epithelium [B] CFTR activity/intestinal tissue [C] Non-CFTR mediated residual chloride secretion/intestinal tissue [D]	[13]
	*CD95 *	ENaC activity/nasal epithelium [A] CFTR activity/nasal epithelium [B] CFTR activity/intestinal tissue [C] Non-CFTR mediated residual chloride secretion/intestinal tissue [D]	[13]
	*IFNGR1 *	CFTR activity/nasal epithelium [B] CFTR activity/intestinal tissue [C]	[13, 39]
	*TLR5 *	CFTR activity/nasal epithelium [B] Non-CFTR mediated residual chloride secretion/intestinal tissue [D]	[13]
	*TLR9 *	CFTR activity/intestinal tissue [C] Non-CFTR mediated residual chloride secretion/intestinal tissue [D]	[13]

Transcription factors	*STAT3 *	CFTR activity/intestinal tissue [C]	[13, 39]
	*EHF *	ENaC activity/nasal epithelium [A] CFTR activity/intestinal tissue [C]	[45]

[A]: the association was observed in a case-reference study comparing F508del-*CFTR* homozygotes with high versus low change of the potential upon superfusion of the nasal epithelium with amiloride as assessed by nasal potential difference measurement. Please see [13] for details.

[B]: the association was observed in a case-reference study comparing F508del-*CFTR* homozygotes with presence versus absence of a change of the potential upon superfusion of the nasal epithelium with chloride-free solution and isoproterenol as assessed by nasal potential difference measurement. Please see [13] for details.

[C]: the association was observed in a case-reference study comparing F508del-*CFTR* homozygotes with presence versus absence of DIDS-insensitive residual chloride secretion as assessed by intestinal current measurement of rectal suction biopsies. Please see [13] for details.

[D]: the association was observed in a case-reference study comparing F508del-*CFTR* homozygotes with presence versus absence of DIDS-sensitive residual chloride secretion of the intestinal tissue as assessed by intestinal current measurement of rectal suction biopsies. Please see [13] for details.

## References

[1] van Meegen M. A., Terheggen-Lagro S. W. J., Koymans K. J., van der Ent C. K., Beekman J. M. (2013). Apical CFTR expression in human nasal epithelium correlates with lung disease in cystic fibrosis. *PLoS ONE*.

[2] Berdiev B. K., Cormet-Boyaka E., Tousson A. (2007). Molecular proximity of cystic fibrosis transmembrane conductance regulator and epithelial sodium channel assessed by fluorescence resonance energy transfer. *The Journal of Biological Chemistry*.

[3] Dobbs L. G., Johnson M. D. (2007). Alveolar epithelial transport in the adult lung. *Respiratory Physiology & Neurobiology*.

[4] Ng A. W., Bidani A., Heming T. A. (2004). Innate host defense of the lung: effects of lung-lining fluid pH. *Lung*.

[5] Hoegger M. J., Fischer A. J., McMenimen J. D. (2014). Impaired mucus detachment disrupts mucociliary transport in a piglet model of cystic fibrosis. *Science*.

[6] Pezzulo A. A., Tang X. X., Hoegger M. J. (2012). Reduced airway surface pH impairs bacterial killing in the porcine cystic fibrosis lung. *Nature*.

[7] Kawai T., Akira S. (2010). The role of pattern-recognition receptors in innate immunity: update on toll-like receptors. *Nature Immunology*.

[8] McClure R., Massari P. (2014). TLR-dependent human mucosal epithelial cell responses to microbial pathogens. *Frontiers in Immunology*.

[9] Panuska J. R., Midulla F., Cirino N. M. (1990). Virus-induced alterations in macrophage production of tumor necrosis factor and prostaglandin E2. *American Journal of Physiology—Lung Cellular and Molecular Physiology*.

[10] Saha B., Jyothi Prasanna S., Chandrasekar B., Nandi D. (2010). Gene modulation and immunoregulatory roles of Interferon *γ*. *Cytokine*.

[11] Levine S. J., Logun C., Chopra D. P., Rhim J. S., Shelhamer J. H. (1996). Protein kinase C, interleukin-1*β*, and corticosteroids regulate shedding of the type I, 55 kDa TNF receptor from human airway epithelial cells. *The American Journal of Respiratory Cell and Molecular Biology*.

[12] Valente G., Ozmen L., Novelli F. (1992). Distribution of interferon-gamma receptor in human tissues. *European Journal of Immunology*.

[13] Stanke F., Becker T., Kumar V. (2011). Genes that determine immunology and inflammation modify the basic defect of impaired ion conductance in cystic fibrosis epithelia. *Journal of Medical Genetics*.

[14] Herold C., Becker T. (2009). Genetic association analysis with FAMHAP: a major program update. *Bioinformatics*.

[15] Frank J., Roux J., Kawakatsu H. (2003). Transforming growth factor-beta1 decreases expression of the epithelial sodium channel alphaENaC and alveolar epithelial vectorial sodium and fluid transport via an ERK1/2-dependent mechanism. *The Journal of Biological Chemistry*.

[16] Galietta L. J. V., Pagesy P., Folli C. (2002). IL-4 is a potent modulator of ion transport in the human bronchial epithelium in vitro. *Journal of Immunology*.

[17] Galietta L. J. V., Folli C., Marchetti C. (2000). Modification of transepithelial ion transport in human cultured bronchial epithelial cells by interferon-gamma. *The American Journal of Physiology—Lung Cellular and Molecular Physiology*.

[18] Roux J., Kawakatsu H., Gartland B. (2005). Interleukin-1*β* decreases expression of the epithelial sodium channel *α*-subunit in alveolar epithelial cells via a p38 MAPK-dependent signaling pathway. *Journal of Biological Chemistry*.

[19] Dagenais A., Fréchette R., Yamagata Y. (2004). Downregulation of ENaC activity and expression by TNF-alpha in alveolar epithelial cells. *The American Journal of Physiology—Lung Cellular and Molecular Physiology*.

[20] Skowron-Zwarg M., Boland S., Caruso N., Coraux C., Marano F., Tournier F. (2007). Interleukin-13 interferes with CFTR and AQP5 expression and localization during human airway epithelial cell differentiation. *Experimental Cell Research*.

[21] Kreindler J. L., Bertrand C. A., Lee R. J. (2009). Interleukin-17A induces bicarbonate secretion in nrmal human bronchial epithelial cells. *The American Journal of Physiology—Lung Cellular and Molecular Physiology*.

[22] Anagnostopoulou P., Dai L., Schatterny J., Hirtz S., Duerr J., Mall M. A. (2010). Allergic airway inflammation induces a pro-secretory epithelial ion transport phenotype in mice. *European Respiratory Journal*.

[23] Anagnostopoulou P., Riederer B., Duerr J. (2012). SLC26A9-mediated chloride secretion prevents mucus obstruction in airway inflammation. *Journal of Clinical Investigation*.

[24] Veit G., Bossard F., Goepp J. (2012). Proinflammatory cytokine secretion is suppressed by TMEM16A or CFTR channel activity in human cystic fibrosis bronchial epithelia. *Molecular Biology of the Cell*.

[25] Peters D. M., Vadász I., Wujak L. (2014). TGF-*β* directs trafficking of the epithelial sodium channel ENaC which has implications for ion and fluid transport in acute lung injury. *Proceedings of the National Academy of Sciences of the United States of America*.

[26] Chang S. S., Grunder S., Hanukoglu A. (1996). Mutations in subunits of the epithelial sodium channel cause salt wasting with hyperkalaemic acidosis, pseudohypoaldosteronism type 1. *Nature Genetics*.

[27] Marthinsen L., Kornfält R., Aili M., Andersson D., Westgren U., Schaedel C. (1998). Recurrent Pseudomonas bronchopneumonia and other symptoms as in cystic fibrosis in a child with type I pseudohypoaldosteronism. *Acta Paediatrica, International Journal of Paediatrics*.

[28] Hanukoglu A., Bistritzer T., Rakover Y., Mandelberg A. (1994). Pseudohypoaldosteronism with increased sweat and saliva electrolyte values and frequent lower respiratory tract infections mimicking cystic fibrosis. *The Journal of Pediatrics*.

[29] Azad A. K., Rauh R., Vermeulen F. (2009). Mutations in the amiloride-sensitive epithelial sodium channel in patients with cystic fibrosis-like disease. *Human Mutation*.

[30] Frank J. A., Matthay M. A. (2014). TGF-*β* and lung fluid balance in ARDS. *Proceedings of the National Academy of Sciences of the United States of America*.

[31] Collaco J. M., Cutting G. R. (2012). Environmental and non-CFTR modifiers of cystic fibrosis. *International Journal of Clinical Reviews*.

[32] Tümmler B., Stanke F., Mall M. A., Elborn J. S. (2014). Genetic and environmental modifiers of cystic fibrosis. *Cystic Fibrosis*.

[33] Kellis M., Wold B., Snyder M. P. (2014). Defining functional DNA elements in the human genome. *Proceedings of the National Academy of Sciences of the United States of America*.

[34] de Vries L., Griffiths A., Armstrong D., Robinson P. J. (2014). Cytokine gene polymorphisms and severity of CF lung disease. *Journal of Cystic Fibrosis*.

[35] Levy H., Murphy A., Zou F. (2009). IL1B polymorphisms modulate cystic fibrosis lung disease. *Pediatric Pulmonology*.

[36] Hillian A. D., Londono D., Dunn J. M. (2008). Modulation of cystic fibrosis lung disease by variants in interleukin-8. *Genes and Immunity*.

[37] Buranawuti K., Boyle M. P., Cheng S. (2007). Variants in mannose-binding lectin and tumour necrosis factor *α* affect survival in cystic fibrosis. *Journal of Medical Genetics*.

[38] Drumm M. L., Konstan M. W., Schluchter M. D. (2005). Genetic modifiers of lung disease in cystic fibrosis. *The New England Journal of Medicine*.

[39] Labenski H., Hedtfeld S., Becker T., Tümmler B., Stanke F. (2011). Initial interrogation, confirmation and fine mapping of modifying genes: STAT3, IL1B and IFNGR1 determine cystic fibrosis disease manifestation. *European Journal of Human Genetics*.

[40] Stanke F., Becker T., Cuppens H. (2006). The TNFalpha receptor TNFRSF1A and genes encoding the amiloride-sensitive sodium channel ENaC as modulators in cystic fibrosis. *Human Genetics*.

[41] Lukacs G. L., Verkman A. S. (2012). CFTR: Folding, misfolding and correcting the ∆F508 conformational defect. *Trends in Molecular Medicine*.

[42] Swiatecka-Urban A., Duhaime M., Coutermarsh B. (2002). PDZ domain interaction controls the endocytic recycling of the cystic fibrosis transmembrane conductance regulator. *The Journal of Biological Chemistry*.

[43] Swiatecka-Urban A., Brown A., Moreau-Marquis S. (2005). The short apical membrane half-life of rescued ΔF508-cystic fibrosis transmembrane conductance regulator (CFTR) results from accelerated endocytosis of ΔF508H-CFTR in polarized human airway epithelial cells. *Journal of Biological Chemistry*.

[44] Ameen N., Silvis M., Bradbury N. A. (2007). Endocytic trafficking of CFTR in health and disease. *Journal of Cystic Fibrosis*.

[45] Stanke F., van Barneveld A., Hedtfeld S., Wölfl S., Becker T., Tümmler B. (2014). The CF-modifying gene EHF promotes p.Phe508del-CFTR residual function by altering protein glycosylation and trafficking in epithelial cells. *European Journal of Human Genetics*.

[46] Wright F. A., Strug L. J., Doshi V. K. (2011). Genome-wide association and linkage identify modifier loci of lung disease severity in cystic fibrosis at 11p13 and 20q13.2. *Nature Genetics*.

